# ABC and MFS efflux transporters mediate antibiotic resistance and bile salt tolerance in *Enterococcus faecalis*

**DOI:** 10.1128/msphere.00489-26

**Published:** 2026-09-15

**Authors:** Q. C. Truong-Bolduc, Y. Wang, S. S. Mello, M. C. Phillips, M. S. Gilmore, D. C. Hooper

**Affiliations:** 1 Infectious Diseases Division and Medical Services, Massachusetts General Hospital, Harvard Medical School 2348, Boston, Massachusetts, USA; 2 Department of Ophthalmology, Mass Eye and Ear, Harvard Medical School 1811 , Boston, Massachusetts, USA; 3 Department of Microbiology, Harvard Medical School 1811 , Boston, Massachusetts, USA; University of Nebraska Medical Center College of Medicine, Omaha, Nebraska, USA

**Keywords:** MFS efflux pump, *E. faecalis*, bile salts, antibiotics, ABC transporter, MFS efflux pump.

## Abstract

Bacteria that reside in the gastrointestinal tract must adapt to the antibacterial properties of bile salts (BSs) and other compounds. We investigated five putative transporters of the enteric-colonizing *Enterococcus faecalis* for their roles in adaptation to BS and antibiotic resistance. Transcript levels of *ef2593* and *ef0575* increased over fivefold, and *ef0420* increased 12-fold when exposed to 0.02% and 0.1% BS concentrations. No change in the expression of *ef2592* or e*f1814* was seen. *E. faecalis* wild-type MMH594 overexpressing efflux pumps by plasmid constructs (EF2592, EF2593, EF0575, EF0420) or by a pre-exposure to 0.02% BS (EF2593 or EF0420) showed a higher CFU/mL than wild type alone when exposed to 0.3% BS, indicating adaptation of *E. faecalis* to resist a higher BS level in the medium, and that *E. faecalis* BS response involved multiple transporters. In MIC assays, the following overexpressors also showed a twofold increase in antibiotic resistance: EF2592 and EF2593 (daptomycin, vancomycin); EF0420 (daptomycin, oritavancin); and EF1814 (daptomycin, levofloxacin, oritavancin, and vancomycin). While no change in EF0575-overexpressor, mutant *0575* showed a significant increase in susceptibility to daptomycin, linezolid, levofloxacin, and oritavancin. In addition, nisin-induced overexpression of several transporters from a plasmid in *Lactococcus lactis* also increased the MICs of multiple antibiotics, most notably for EF1814, with a 4- to 16-fold increase in the MICs of daptomycin, levofloxacin, linezolid, and vancomycin. These data indicate that expression of multiple *E. faecalis* transporters responds to bile salts and may contribute to survival and colonization in the gut, as well as causing low-level multidrug resistance.

Bile salts are key antimicrobial components of the gastrointestinal tract, and bacterial adaptation to bile salt stress is critical for host colonization and persistence. In this study, we demonstrate that multiple *E. faecalis* transporters are induced by bile salts and contribute to adaptation at physiologically relevant bile salt concentrations. Importantly, several of these transporters also confer low-level resistance to clinically important antibiotics, including daptomycin, vancomycin, levofloxacin, linezolid, and oritavancin. These findings suggest that transport systems selected for survival in the intestinal environment may have the unintended consequence of increasing antibiotic tolerance and multidrug resistance. By linking host-associated environmental stress with antimicrobial resistance phenotypes, this work advances our understanding of how gut colonization factors can influence bacterial persistence and therapeutic outcomes. The identified transporters represent potential targets for strategies to limit both intestinal colonization and antibiotic resistance in *E. faecalis*.

## INTRODUCTION

*Enterococcus faecalis* is a commensal gram-positive bacterium that naturally inhabits the gastrointestinal tract of humans, and certain strains have been used as probiotics (1). *E. faecalis* is capable of living in various environments due to its highly adaptive genome, easily acquiring and transferring genes across species, resulting in resistance to multiple antibiotics and drug compounds (2, 3). Despite its commensal role, *E. faecalis* also thrives as a nosocomial pathogen in healthcare settings, causing life-threatening diseases, especially in hospital-acquired infections, due to an increase in multidrug-resistant (MDR) *E. faecalis* strains (4, 5).

*E. faecalis* is intrinsically resistant to the majority of cephalosporins, except for ceftaroline and ceftobiprole, which are active against *E. faecalis in vitro* (5). In addition, acquired resistance to tetracyclines, aminoglycosides, and vancomycin is widespread in *E. faecalis*, and the minimum inhibitory concentration (MIC) of ciprofloxacin indicates borderline activity for non-urinary tract infections (6). This pathogen is usually susceptible to ampicillin, linezolid, daptomycin, and oritavancin (5, 7, 8). While the mechanisms underlying antibiotic resistance are well described, the roles of *E. faecalis* MDR efflux pumps and their contributions to environmental adaptation and drug resistance are still incomplete.

Studies have evaluated genes encoding potential transporters of *E. faecalis* V583, including efflux pumps of the ATP-binding cassette (ABC) and the major facilitator superfamily (MFS) superfamilies (9), such as the chromosomal ABC transporter LsaA (EF2720), responsible for the intrinsic resistance of *E. faecalis* to quinupristin-dalfopristin (10). OptrA, a plasmid-expressed homolog of LsaA, was also reported to confer resistance to linezolid in enterococci (11). Other heterodimeric ABC transporters have been found associated with *E. faecalis* resistance to doxorubicin, ethidium bromide, quinolones, or chlorhexidine, including EfrAB (EF2920–EF2919) (12), EfrCD (EF0789-EF0790) (13), and EfrEF (14). The *E. faecalis* MFS efflux pump EmeA (EF1078) is a homolog of the *Staphylococcus aureus* NorA multidrug resistance efflux pump, and EmeA is also responsible for *E. faecalis* resistance to quinolones (ciprofloxacin, norfloxacin) and ethidium bromide (15, 16).

As a colonizer of the human intestinal tract, *E. faecalis* is exposed to bile acids and their salts, predominantly cholic acid (CA; 40%) and chenodeoxycholic acid (CDCA; 40%), the secondary bile acid, deoxycholic acid (DCA; 20%), and a minor level of lithocholic acid (17). Bile salts (BSs) act as biological detergents and specifically alter the lipid and fatty acid composition of bacterial cell membranes, increasing membrane permeability and leading to leakage of intracellular materials. High concentrations of BS lead to the dissolution of membrane lipids and the dissociation of membrane proteins, resulting in bacterial cell death (18). The transcriptional response of *E. faecalis* to BS, sodium dodecyl sulfate (SDS), and (BS + SDS) exposures has been analyzed using transcriptomic data, and showed that genes encoding fatty acids and phospholipid metabolism were repressed, while genes encoding MDR transporters and V-type ATPases were induced (19).

In this study, we investigated five putative transporters of *E. faecalis* that might play a role in *E. faecalis* survival in the presence of BS and antibiotic resistance. We selected two putative exporters, EF2592 and EF2593, identified from the proteomic response data of *E. faecalis* V583 exposed to BS (CA, CDCA, DCA), and showed homology to the doxorubicin-resistance ABC transporters EfrD and EfrC, respectively (13, 17, 20). We also selected the ABC importer EF0575 from a high-complexity mariner transposon insertion library in *E. faecalis* MMH594 (21) that showed homology to the ATP-binding protein LsaA and was part of an EfaR-regulated operon, which was upregulated in the presence of high levels of metal ions. EfaR is an important virulence metalloregulator that modulates the manganese-dependent pathogenicity of *E. faecalis* (22). We included two putative MFS efflux pumps, EF0420 and EF1814, identified from the transcriptomic data of *E. faecalis* V583 grown in the presence of bovine BS and sodium dodecyl sulfate (19). EF0420 and EF1814 showed homology to the EmrB/QacA family of MDR efflux pumps, a member of which, EmrB, was previously reported to participate in the BS efflux of *Escherichia coli* (23).

We demonstrated that, when overexpressed, EF2592, EF2593, EF0575, and EF0420 improved *E. faecalis* survival in the presence of BS, and that *E. faecalis* adapted to a sub-lethal level of BS (0.02%) survived better than non-adapted *E. faecalis* when challenged with 0.3% BS, an assay previously used to investigate *E. faecium* resistance to BS (24). In addition, four over-expressed transporters caused twofold increases in the antimicrobial MICs, including daptomycin and vancomycin (EF2592, EF2593), daptomycin and oritavancin (EF0420), and daptomycin, levofloxacin, oritavancin, and vancomycin (EF1814). Furthermore, overexpressors of EF2593 and EF0420, when co-exposed to 0.02% or 0.1% BS + antibiotics, showed an additional twofold increase in the MICs of levofloxacin, linezolid, and vancomycin. We also overexpressed the selected transporters in *Lactococcus lactis* NZ9000 to confirm their roles in antibiotic resistance. We found a 4- to 16-fold increase in the MICs of the MFS efflux pump EF1814 of the tested antibiotics, while other transporters also showed at least a twofold increase in the MICs, including linezolid. In contrast, only the *mariner* insertion mutant MMH594*ef0575::mariner* exhibited an increase in susceptibility to antibiotics, which was reversed by complementation with a plasmid-borne EF0575.

## RESULTS

### Putative transporters showed homology to the ABC transporter EfrCD

**Fig 1:**
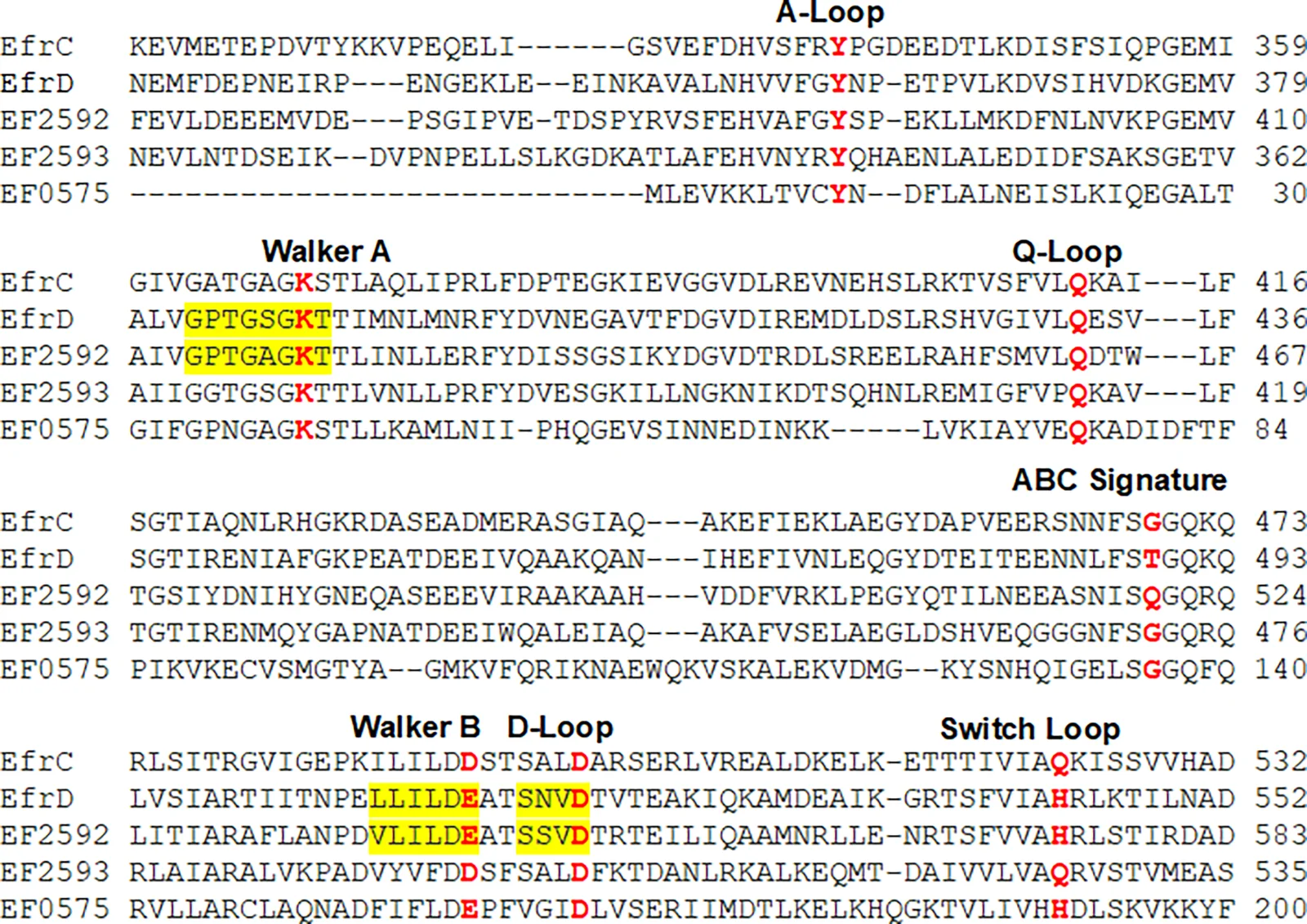
Putative ABC transporters of *E. faecalis*. Multiple sequence alignment of the ATP-binding domains of EF2592, EF2593, and EF0575 using ClustalW and sequences from the NCBI website. EF2592, EF2593, and EF0575 were compared with EfrC and EfrD amino acid sequences. The seven conserved motifs of the NBD of ABC transporters, A-loop, Walker A, Q-loop, ABC signature, Walker B, D-loop, and Switch loop are indicated, and the conserved amino acids are shown in red.

EF2592 and EF2593 showed, respectively, 30% and 35% identity to EfrC, and respectively, 37% and 30% identity to EfrD. The ABC transporter EfrCD confers enterococcal resistance to doxorubicin, daunorubicin, and ethidium bromide when overexpressed in *L. lactis* (13, 25, 26). We performed BLAST analysis (27) and Clustal Omega Multiple Sequence Alignments (https://www.ebi.ac.uk/jdispatcher/msa/clustalo) (28) using the published amino acid sequences of EF2592, EF2593, and EF0575 of *E. faecalis* V583 (29). EF2592 and EF2593 are separate monomeric ABC transporters that showed structural characteristics of proteins of the ABC transporter superfamily, which included a transmembrane domain (TMD) and a nucleotide-binding domain (NBD) (13, 30, 31). Predicted structures of EF2592 and EF2593 (https://www.sbg.bio.ic.ac.uk/~phyre2/) (32) showed that the nucleotide-binding domain (NBD) of EF2592 and EF2593 shared the same conserved amino acids of motifs essential for the NBD dimerization of the *E. faecalis* ABC transporters EfrD and EfrC, respectively (Fig. 1). The ABC-signature conserved residue threonine (T, EfrD) was replaced by a glutamine (Q) in EF2592 and a glycine (G) in EF2593 (25).

### *E. faecalis* ABC and MFS transporters are induced by BS exposure

We evaluated the transcript levels of five candidate transporters (EF2592, EF2593, EF0420, EF1814, and EF0575) of *E. faecalis* MMH594 for response to BS.

**Fig 2:**
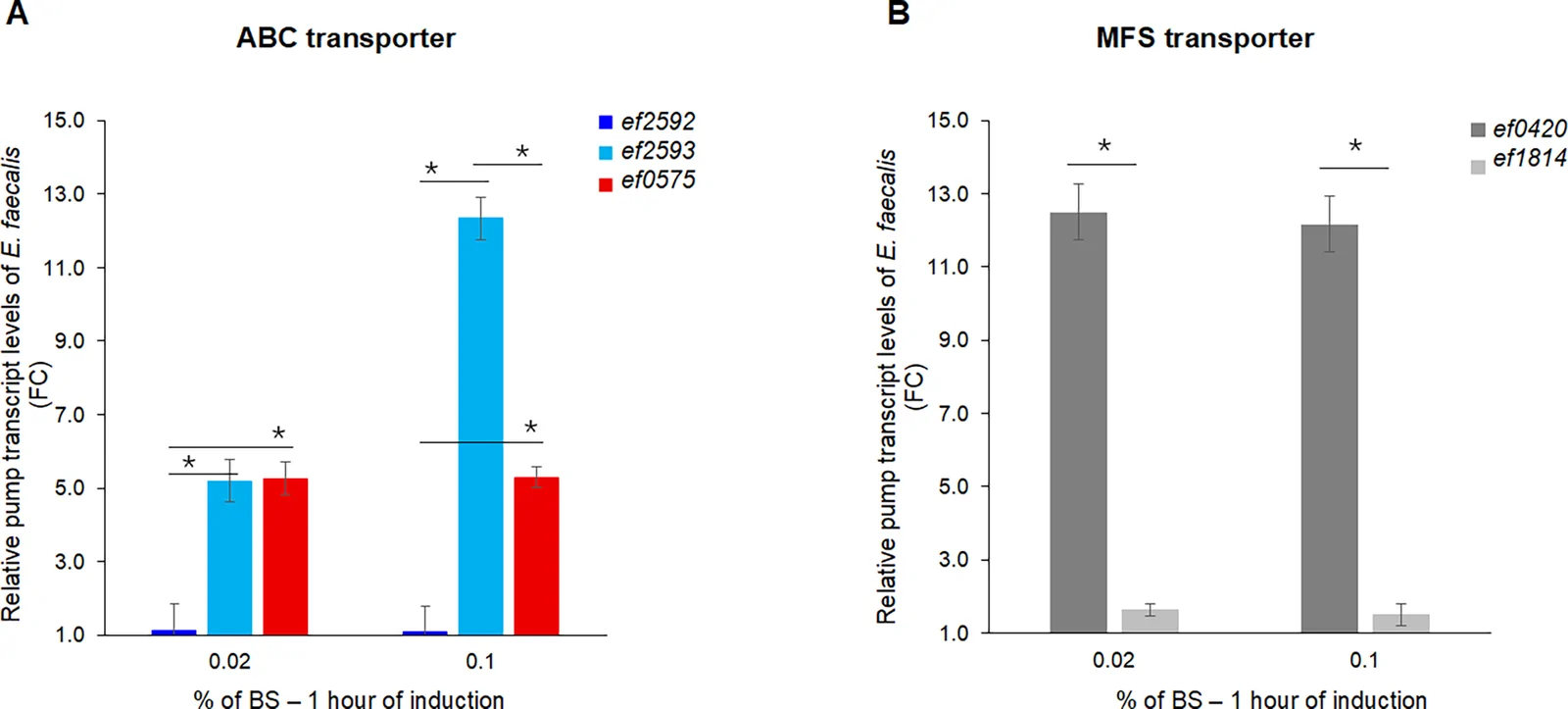
Relative transcript levels of *E. faecalis* ABC transporters and MFS efflux pumps exposed to 0.02% and 0.1% BS. The relative transcript levels of *ef2592, ef2593,* and *ef0575* (**A**) and *ef0420,* and *ef1814* (**B**) were expressed as the fold change (FC) in gene transcripts of *E. faecalis* MMH594 grown in BHI supplemented with 0.02% or 0.1% BS vs BHI alone. Values were normalized against MMH594 in BHI without BS. The assays were repeated three times with three independent biological samples. The error bars represent the means of FC ± SEM for each assay. The * describes the differences in the FC of the ABC transporters or MFS efflux pumps. The differences were statistically significant as determined by an ANOVA (*P* < 0.05). BS, bile salt.

Exposure to BS at sub-lethal concentrations (BS at 0.02% and 0.1%; MIC_BS_ of MMH594 = 0.125%) (17, 24) increased the transcript levels of *ef2593* (5.2-fold at 0.02%; 12.5-fold at 0.1%), *ef0575* (5.3-fold at 0.02% and 0.1%) (Fig. 2A), and *ef0420* (12.5-fold at 0.02%; 12.2-fold at 0.1%) (Fig. 2B), when compared with MMH594 without exposure and after 1 h of induction. In contrast, the transcript levels of *ef2592* and *ef1814* showed no significant increase at both 0.02% and 0.1% BS (Fig. 2A and B).

Although *ef2592* and *ef2593* are encoded within the same operon, only *ef2593* exhibited statistically significant transcript induction under the conditions tested. This difference may reflect differential transcript stability or processing rather than independent transcriptional regulation (Fig. 2A).

### ABC and MFS transporters mediate *E. faecalis* improved survival with BS stress

**Fig 3:**
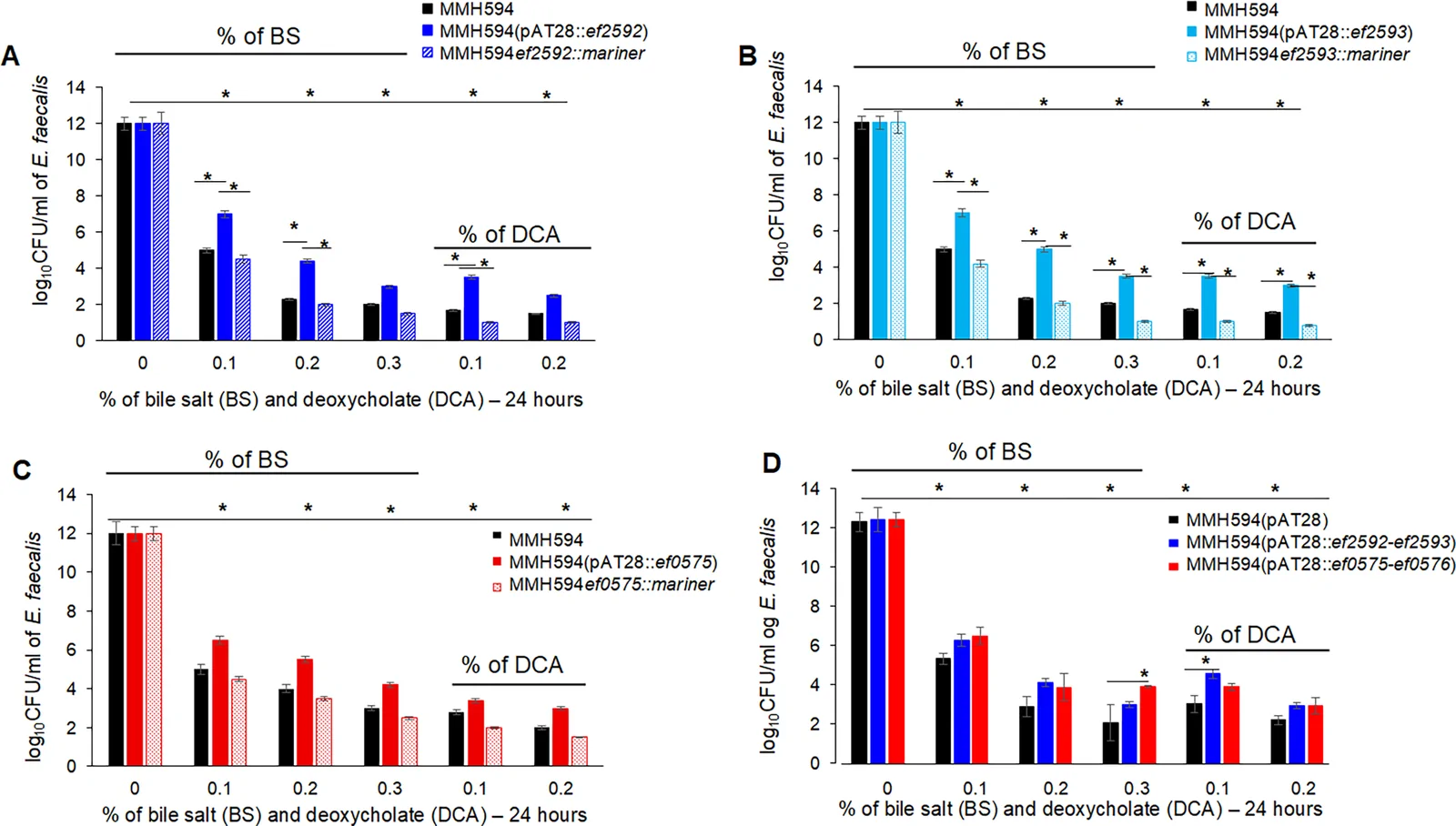
Roles of transporters in *E. faecalis* survival after 24 h exposed to BS or DCA. Growth of *E. faecalis* wild-type MMH594, mutants, and overexpressors after 24 h in the presence of BS at 0%, 0.1%, 0.2%, and 0.3%, or DCA at 0.1% and 0.2%. The initial bacteria before adding BS were 2.2 × 10^8^ CFU/mL, and the value on the Y-axis is represented as log_10_ CFU/mL. (**A**) *E.faecalis* mutant or overexpressor of the ABC transporter EF2592; (**B**) *E. faecalis* mutant or overexpressor of the ABC transporter EF2593; (**C**) *E. faecalis* mutant or overexpressor of the ABC transporter EF0575; (**D**) *E. faecalis* double overexpressors of EF2592-EF2593 or EF0575-EF0576. EF0576 is the predicted permease of the putative heterodimeric ABC transporter EF0575-EF0576. The transcript levels of *ef2592, ef2593, ef0575*, and *ef0576* increased fourfold as determined by real-time RT-PCR. The assays were repeated three times with three different biological samples. The error bars represent the means of log_10_ CFU/mL ± SD for each assay. The * represents the differences in log_10_ CFU/mL between wild-type MMH594, overexpressors, and mutants, exposed to BS or DCA versus non-exposed. The differences were statistically significant as determined by an ANOVA (*P < 0.05*). MMH594 and MMH594(pAT28) showed the same growth pattern in BHI and BHI plus BS. BS, bile salt; DCA, deoxycholate.

At 0.1% and 0.2% BS exposure for 1 h, overexpression of transporters using plasmid pAT28 caused increased survival for the following pump-overexpressors: MMH594(pAT28::*ef2592*) (2 log_10_ CFU/mL), MMH594(pAT28::*ef2593*) (2 log_10_ and 2.5 log_10_ CFU/mL), and MMH594(pAT28::*ef0575*) (1.5 log_10_ CFU/mL), compared with MMH594(pAT28) that carried the plasmid alone (Fig. 3A through C).

At 0.3% BS (similar to BS concentration in the human intestine) (33) and DCA (0.1%–0.2%), we observed a modest increase in survival for MMH594(pAT28::*ef2592*), MMH594(pAT28::*ef2593*), and MMH594(pAT28::*ef0575*) (1.5 log_10_ CFU/mL), compared with MMH594(pAT28).

Plasmid-mediated overexpression of either *ef2592, ef2593,* or *ef0575* in the wild-type MMH594 background increased bile salt tolerance. Because these experiments were performed in the presence of the endogenous chromosomal *ef2592-ef2593* and *ef0575* copies, these phenotypes are most consistent with increased formation of functional heterodimeric ABC transporters rather than independent activity of individual half-transporters.

Since EF2592-EF2593 (predicted type IV ABC transporter) and EF0575-EF0576 (predicted type II ABC transporters) have separately encoded ATPase and permease proteins, as was previously described for EfrCD (EF0789-EF0790) (13, 20, 25), we evaluated the effect of a dual overexpression of EF2592-EF2593 or EF0575-EF0576 on *E. faecalis* MMH594 growth in similar conditions. *E. faecalis* with a dual expression, MMH594(pAT28::*ef2592-ef2593*) and MMH594(pAT28::*ef0575-ef0576*), also showed an increase in survival (1.5 to 2 log_10_ CFU/mL) as was observed in single overexpression when exposed to BS or DCA, and compared with MMH594(pAT28) (Fig. 3D).

In contrast, *E. faecalis* MMH594 *mariner* insertion mutants MMH594*ef2592::mariner* (Fig. 3A), MMH594*ef2593::mariner* (Fig. 3B), and MMH594*ef0575::mariner* (Fig. 3C), showed ~1-log_10_ CFU/mL decrease in survival when exposed to all concentrations of BS and DCA. As observed with *ef0575*, the transcript level of *ef0576* also increased fivefold at 0.02% and 0.1% BS (data not shown).

**Fig 4:**
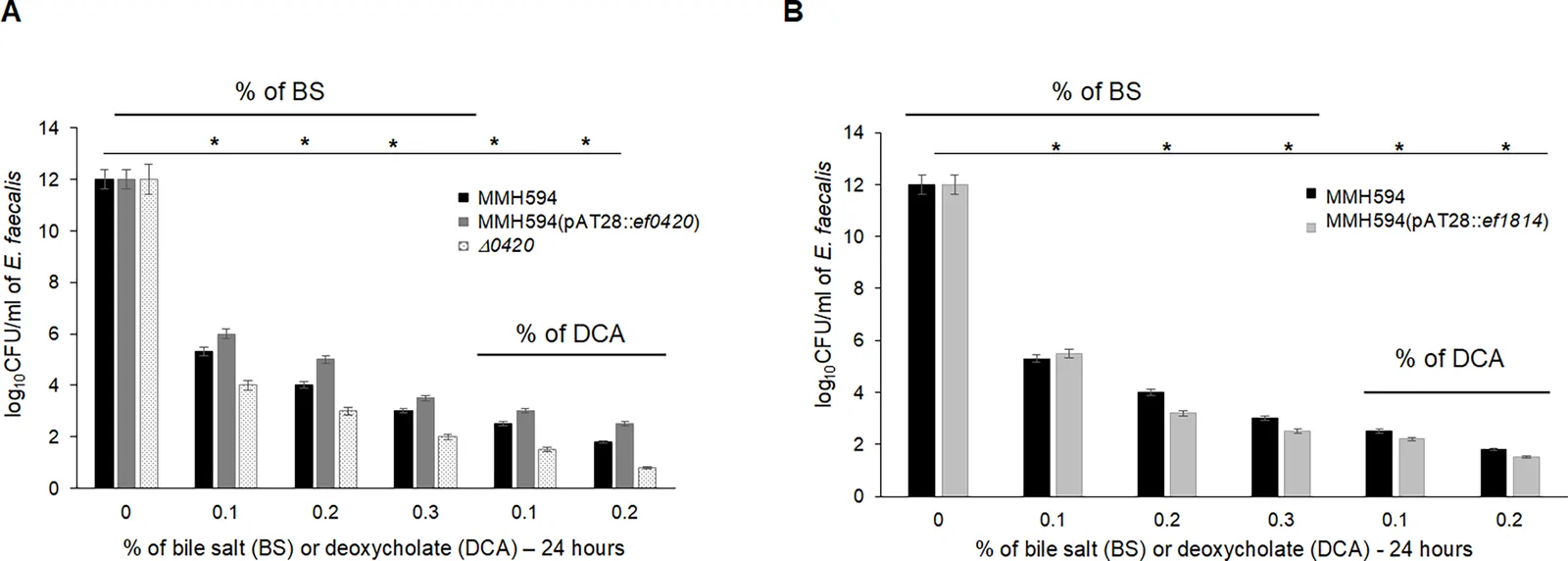
Roles of EF0420 and EF1814 in *E. faecalis* survival after 24 h exposed to BS or DCA. Growth of *E. faecalis* wild-type MMH594, mutant Δ*0420*, and overexpressors after 24 h in the presence of BS at 0%, 0.1%, 0.2%, and 0.3%, or DCA at 0.1% and 0.2%. The initial bacteria before adding BS were 2.2 × 10^8^ CFU/mL, and the value on the *Y*-axis is represented as log_10_ CFU/mL. (**A**) *E.faecalis* mutant Δ*0420* or overexpressor MMH594(pAT28::*ef0420*) of the MFS efflux pump EF0420. (**B**) *E. faecalis* overexpressor MMH594(pAT28::*ef1814*) of the MFS efflux pump EF1814. The assays were repeated three times with three different biological samples. The error bars represent the means of log_10_ CFU/mL ± SD for each assay. The * represents the differences in log_10_ CFU/mL between wild-type MMH594, mutant Δ*0420*, and the overexpressor MMH(pAT28::*ef0420*) or MMH(pAT28::*ef1814*), exposed to BS or DCA versus non-exposed. The differences were statistically significant as determined by an ANOVA (*P* < 0.05). MMH594, MMH(pAT28) showed the same growth pattern in BHI and BHI + BS. BS, bile salt; DCA, deoxycholate.

At BS (0.1%–0.3%) and DCA (0.1%–0.2%), pump-overexpressor MMH594(pAT28::*ef0420*) (Fig. 4A) showed a small increase in survival (<1 log_10_ CFU/mL), and pump-overexpressor MMH594(pAT28::*ef1814*) (Fig. 4B) was less fit to survive at BS concentrations higher than 0.1%, compared with MMH594(pAT28). In addition, the deletion mutant Δ*0420* (Fig. 4A) showed a 1.5-log_10_ CFU/mL decrease in survival under the same conditions.

### ABC and MFS transporters confer antibiotic resistance in *E. faecalis* and *L. lactis*

We carried out MIC testing to determine the effects of *E. faecalis* transporters on susceptibility to antibiotics, including daptomycin and vancomycin, which are commonly used to treat *E. faecalis* infections.

The *E. faecalis* single overexpressors MMH594(pAT28::*ef2592*) and MMH594(pAT28::*ef2593*) showed a twofold increase in the MICs of daptomycin and vancomycin. In contrast, the dual overexpressor MMH594(pAT28::*ef2592-ef2593*) showed an additional twofold increase in the MIC of oritavancin (Table 1).

**TABLE 1:** MICs of *E. faecalis* mutants and overexpressors of efflux transporters

Strain	MIC (µg/mL)^*a*^				
	Daptomycin	Levofloxacin	Linezolid	Oritavancin	Vancomycin
*E. faecalis*					
MMH594	2	0.5	1.25	0.12	0.5
MMH594(pAT28)	2	0.5	1.25	0.12	0.5
ABC transporters					
MMH594(pAT28::*ef2592*)	**4**	0.5	1.25	0.12	**1**
MMH594*ef2592::mariner*	2	0.5	1.25	0.12	0.5
MMH594(pAT28::*ef2593*)	**4**	0.5	1.25	0.12	**1**
MMH594*ef2593::mariner*	2	0.5	1.25	0.12	0.5
MMH594(pAT28::*ef2592-ef2593*)	**4**	0.5	1.25	**0.25**	**1**
MMH594(pAT28::*ef0575*)	2	0.5	1.25	0.12	0.5
MMH594*ef0575::mariner*	0.03	0.06	0.08	0.06	0.5
MMH594*ef0575::mariner*(pAT28::*ef0575*)	2	0.5	1.25	0.12	0.5
MMH594(pAT28::*ef0575-ef0576*)	2	0.5	1.25	**0.25**	0.5
MFS transporters					
MMH594(pAT28::*ef0420*)	**4**	0.5	1.25	**0.25**	0.5
Δ*0420*	2	0.5	1.25	0.12	0.5
MMH594(pAT28::*ef1814*)	**4**	**1**	1.25	**0.25**	**1**

^*a*^ Bold, increase in MIC.

MMH594(pAT28::*ef0420*) and MMH594(pAT28::*ef1814*) both showed a twofold increase in the MICs of daptomycin and oritavancin, with MMH594(pAT28::*efEF1814*) also causing a twofold increase in the MICs of levofloxacin and vancomycin (Table 1).

The MICs of MMH594(pAT28::*ef0575*) were similar to those of wild-type MMH594(pAT28), and the dual overexpressor MMH594(pAT28::*ef0575-ef0576*) showed a twofold increase in the MIC of oritavancin (Table 1). Attempts to generate an EF0576-overexpressor alone were unsuccessful. qRT-PCR demonstrated a fourfold increase in *ef0575* transcripts in MMH594(pAT28::*ef0575*) compared with that of MMH594(pAT28) (data not shown). These data suggested that EF0575 was transcribed but may not be functional alone.

Since our attempts to transform MMH594 with pAT28 plasmids carrying *ef420 and ef1814* were unsuccessful, we transformed *E. faecalis* OG1RF (34) with plasmid constructs of plasmid pMSP3535 carrying *ef0420* and *ef1814*. The pump-overexpressor OG1RF(pMSP3535::*ef0420*) showed a four- and twofold increase in the MICs of daptomycin and levofloxacin, respectively. The pump-overexpressor OG1RF(pMSP3535::*ef1814*) showed an eight-, four-, and twofold increase in the MICs of daptomycin, levofloxacin, and vancomycin, respectively (Table 2).

**TABLE 2:** Phenotypic analysis of wild-type, overexpression, and mutant strains supports the requirement for both subunits of the putative heterodimeric ABC transporters EF2592-EF2593^*a*^

*E. faecalis*	FC		MIC (µg/mL)			log_10_ CFU/mL (24 h)	
	ef2592	ef2593	DAP	ORI	VAN	BS (0.3%)	DCA (0.2%)
MMH594^*b*^	1 ± 0.01	1 ± 0.01	2	0.12	0.5	2 ± 0.1	1.1 ± 0.1
MMH594(pAT28)^*c*^	1 ± 0.01	1 ± 0.01	2	0.12	0.5	3 ± 0.1	2.3 ± 0.2
MMH594(pAT28::*ef2592-ef2593*)	**8.5 ± 0.02**	**10 ± 0.03**	**4**	**0.25**	**1**	3 ± 0.2	3.1 ± 0.1
MMH594(pAT28::*ef2592*)	**8.2 ± 0.01**	1.8 ± 0.01	**4**	0.12	**1**	3 ± 0.1	2.5 ± 0.3
MMH594*ef2592*::mariner		1.8 ± 0.01	2	0.12	0.5	1.5 ± 0.1	1 ± 0.1
MMH594*ef2592*::mariner(pAT28::*ef2592*)	**7.5 ± 0.01**	1.7 ± 0.01	**4**	0.12	**1**	3 ± 0.1	2.1 ± 0.1
MMH594*ef2592*::mariner(pAT28::*ef2593*)		**10.5 ± 0.02**	2	0.12	0.5	1.2 ± 0.1	0.9 ± 0.1
MMH594(pAT28::*ef2593*)	1 ± 0.01	**10 ± 0.02**	**4**	0.12	**1**	3.5 ± 0.1	3 ± 0.1
MMH594*ef2593*::mariner	0.9 ± 0.01		2	0.12	0.5	1 ± 0.1	0.8 ± 0.1
MMH594*ef2593*::mariner(pAT28::*ef2592*)	**8.5 ± 0.01**		2	0.12	0.5	1.1 ± 0.2	0.75 ± 0.1
MMH594*ef2593*::mariner(pAT28::*ef2593*)	1.1 ± 0.01	**8.2 ± 0.02**	**4**	0.12	**1**	3.2 ± 0.1	2.5 ± 0.1

^*a*^ FC, relative gene transcript level in fold change; no induction by bile salts. DAP, daptomycin; ORI, oritavanxin; VAN, vancomycin. Bold, increase in gene FC or MICs of antibiotics**.**

^*b*^ Reference for *E. faecalis* MMH594 and mutants.

^*c*^ Reference for MMH594 and mutants pump-overexpressors.

We also performed MIC testing on transporter mutants MMH594*ef2592::mariner*, MMH594*ef2593::mariner*, MMH594*ef0575::mariner*, and the deletion mutant Δ*0420* to assess the effect of transporters on *E. faecalis* susceptibility to antibiotics. Our attempts to create a deletion mutant Δ*1814* were unsuccessful. The mutant MMH594*ef0575::mariner* was cultured in the presence of tetracycline at 5 µg/mL as a selection pressure, as previously described (21) (MIC_TETRA_ of MMH594*ef0575::mariner* = 7.5 µg/mL). All mutants except MMH594*ef0575::mariner* showed MICs similar to those of MMH594 (Table 1). Notably, mutant MMH594*ef0575::mariner* showed an increase in susceptibility of 66-fold to daptomycin, 8-fold to levofloxacin, 15.6-fold to linezolid, and 2.1-fold to oritavancin. Complementation of MMH594*ef0575::mariner* with the plasmid construct pAT28::*ef0575* restored the MICs to values comparable to those of MMH594 (Table 1). Verification by qRT-PCR showed an eightfold increase in the *ef0575* transcript level in MMH594*ef0575::mariner*(pAT28::*ef0575*) compared with that in MMH594.

Overexpression of *ef2593* in MMH594*ef2592::mariner* did not increase daptomycin or vancomycin MICs and failed to restore bile salt or deoxycholate tolerance. Similarly, overexpression of *ef2592* in MMH594*ef2593::mariner* did not confer these phenotypes.

In contrast, overexpression of each gene restored the phenotype in its corresponding mutant background, confirming the functionality of the plasmid constructs.

The qRT-PCR analysis further demonstrated minimal cross-induction between *ef2592* and *ef2593*, indicating that overexpression of one gene did not substantially alter transcription of its predicted partner (Table 2).

To evaluate and confirm the resistance properties of the five transporters independently of their expression in the endogenous context of *E. faecalis*, EF2592, EF2593, EF0575-0576, EF0420, and EF1814 were overexpressed in *L. lactis* NZ9000 (13). Attempts to transform NZ9000 with plasmid construct (pMSP3535::*ef0575*) were unsuccessful. We induced the overexpression of *E. faecalis* transporters by adding 25 ng/mL of nisin to the NZ9000 cultures as previously described (35), and performed MICs.

The MICs of *L. lactis* pump overexpressors NZ9000(pMSP3535::*ef2592*) and NZ9000(pMSP3535::*ef2593*) showed a fourfold increase for daptomycin and a twofold increase for levofloxacin, linezolid, and oritavancin (Table 3). The dual overexpressor NZ9000(pMSP3535::*ef0575-ef0576*) showed a twofold increase in the MIC of daptomycin, while no change occurred in the MICs of NZ9000(pMSP3535::*ef0576*). The MICs of pump-overexpressor NZ9000 (pMSP3535::*ef0420*) showed a fourfold increase in daptomycin. In contrast, the pump-overexpressor NZ9000(pMSP3535::*ef1814*) showed a 16-fold increase in daptomycin and levofloxacin, a fourfold increase in linezolid, and an eightfold increase in vancomycin (Table 3). These results confirmed the ability of these pumps to confer low- to moderate resistance to several antibiotics when overexpressed from plasmids.

**TABLE 3:** MICs of *L. lactis* NZ9000 and *E. faecalis* OG1RF overexpressing *E. faecalis* efflux transporters

Strain	MIC (µg/mL)^*a*^				
	Daptomycin	Levofloxacin	Linezolid	Oritavancin	Vancomycin
*L. lactis*					
NZ9000	0.5	0.5	0.31	0.06	0.25
NZ9000(pMSP3535) ± nisin	0.5	0.5	0.31	0.06	0.25
ABC transporters ± nisin^*b*^					
NZ9000(pMSP3535::*ef2592*)	**2**	**2**	**1.25**	**0.12**	0.03
NZ9000(pMSP3535::*ef2593*)	**2**	**2**	**1.25**	**0.12**	0.03
NZ9000(pMSP3535::*ef0576*)	0.5	0.5	0.625	0.06	0.03
NZ9000(pMSP3535::*ef0575-ef0576*)	**1**	1	0.625	0.06	0.03
MFS transporters without nisin					
NZ9000(pMSP3535)	0.5	0.5	0.31	0.06	0.25
NZ9000(pMSP3535::*ef0420*)	**1**	0.5	0.31	0.06	0.25
NZ9000(pMSP3535::*ef1814*)	**2**	**1**	**0.62**	0.06	**1**
MFS transporters with nisin					
NZ9000(pMSP3535)	0.5	0.5	0.31	0.06	0.25
NZ9000(pMSP3535::*ef0420*)	**2**	0.5	0.31	0.06	**1**
NZ9000(pMSP3535::*ef1814*)	**8**	**8**	**1.25**	0.06	**2**
*E. faecalis*					
OG1RF	2	0.5	1.25	0.12	0.5
OG1RF(pMSP3535)	2	0.5	1.25	0.12	0.5
MFS transporters without nisin					
OG1RF(pMSP3535)	2	0.5	1.25	0.12	0.5
OG1RF(pMSP3535::*ef0420*)	**4**	0.5	1.25	0.12	0.5
OG1RF(pMSP3535::*ef1814*)	**4**	**1**	1.25	0.12	**1**
MFS transporters with nisin					
OG1RF(pMSP3535)	2	0.5	1.25	0.12	0.5
OG1RF(pMSP3535::*ef0420*)	**8**	**1**	1.25	0.12	0.5
OG1RF(pMSP3535::*ef1814*)	**16**	**2**	1.25	0.12	**1**

^*a*^ Bold, increase in MICs.

^*b*^ Nisin at 25 ng/mL.

### Co-exposure of 0.02% or 0.1% BS + antibiotics increases *E. faecalis* MICs

We determined if exposure to a sub-lethal concentration of BS would increase *E. faecalis* MMH594 resistance to antibiotics (MIC_BS_ of MMH594 = 0.125%). MICs carried out with BS at 0.02% or 0.1% ± antibiotics showed that the pump-overexpressors MMH594(pMSP3535::*ef2593*) and MMH594(pMSP3535::*ef0420*) exhibited an additional twofold increase in the MICs of levofloxacin and linezolid, and a fourfold increase in the MIC of vancomycin, compared with the MICs of antibiotics without BS (Table 4).

**TABLE 4:** MICs of *E. faecalis* overexpressing efflux transporters with or without BS at 0.02% and 0.1%

*E. faecalis*	MIC (μg/mL)^*a*^				
	Daptomycin	Levofloxacin	Linezolid	Oritavancin	Vancomycin
Antibiotics alone					
MMH594(pAT28)	2	0.5	1.25	0.12	0.5
MMH594(pAT28::*ef2593*)	4	0.5	1.25	0.12	1
MMH594(pAT28::*ef0420*)	4	0.5	1.25	0.25	0.5
MMH594(pAT28::*ef1814*)	4	1	1.25	0.25	1
Antibiotics + BS (0.02%)					
MMH594(pAT28)	2	0.5	1.25	0.12	0.5
MMH594(pAT28::*ef2593*)	4	**1**	**2.5**	0.12	**2**
MMH594(pAT28::*ef0420*)	4	**1**	**2.5**	0.25	**2**
MMH594(pAT28::*ef1814*)	4	1	**1.5**	0.25	**2**
Antibiotics + BS (0.1%)					
MMH594(pAT28)	2	0.5	1.25	0.12	0.5
MMH594(pAT28::*ef2593*)	4	**1**	**2.5**	0.12	**2**
MMH594(pAT28::*ef0420*)	4	**1**	**2.5**	0.25	**2**
MMH594(pAT28::*ef1814*)	4	1	**1.5**	0.25	**2**

^*a*^ Bold, additional increase in MIC.

The pump-overexpressor MMH594(pMSP3535::*ef1814*) showed a slight increase in the MIC of linezolid (from 1.25 to 1.5 μg/mL) and a twofold increase in vancomycin MIC (Table 4).

### Pre-exposure with 0.02% BS improved *E. faecalis* survival against 0.3% BS or antibiotics

**Fig 5:**
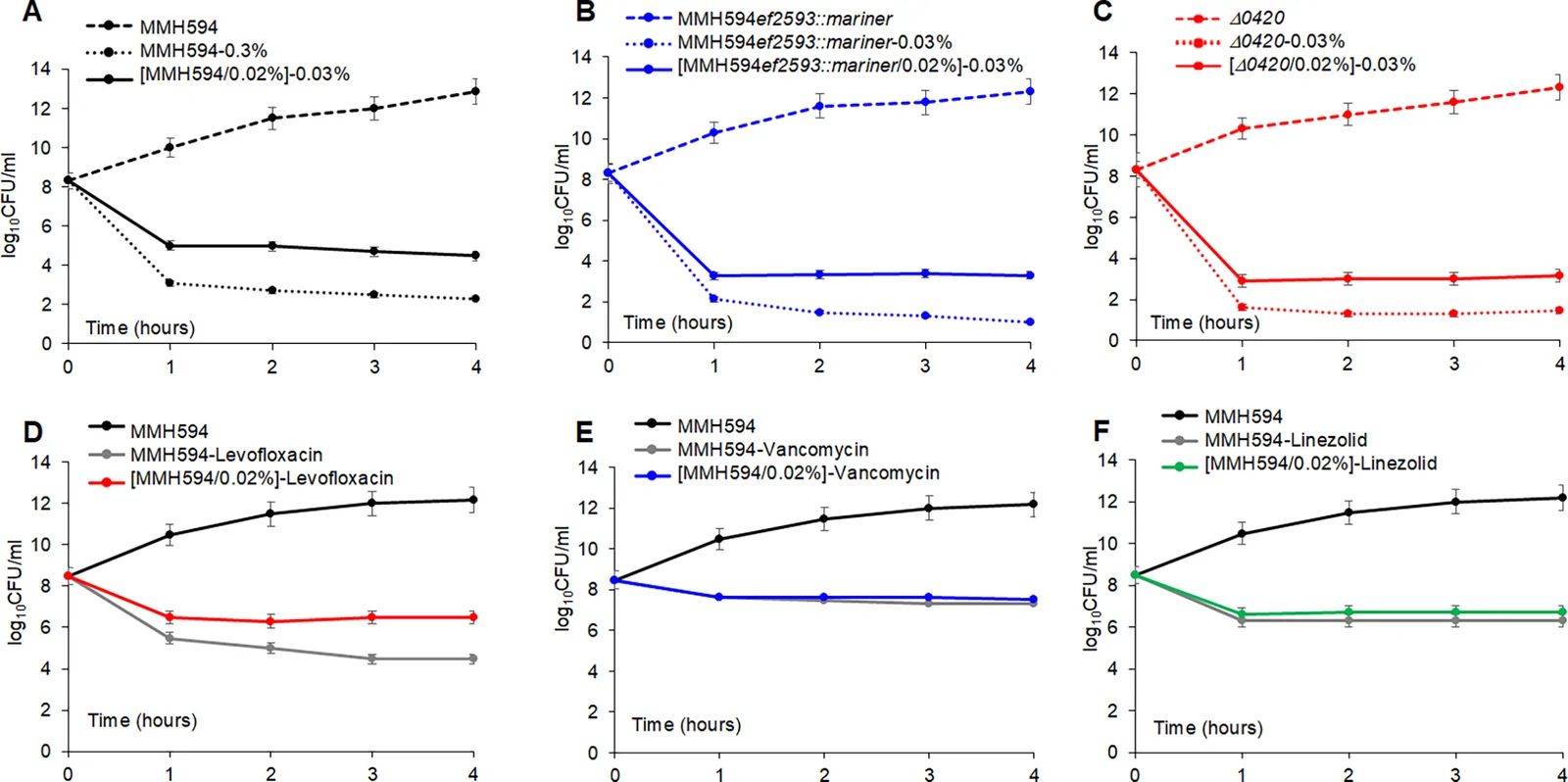
Survival of *E. faecalis* following exposure to 0.3% BS or antibiotics with and without pre-adaptation with 0.02% BS. (**A–C**) *E. faecalis* MMH594 (**A**), mutant MMH594*ef2593::mariner* (**B**), and mutant Δ*0420* (**C**) were exposed to 0.02% BS for 15 min and then challenged with 0.3% BS for 4 h. (**D–F**) *E. faecalis* MMH594 was exposed to 0.02% BS for 15 min and then challenged with 3× MIC of levofloxacin (**D**), vancomycin (**E**), or linezolid (**F**) for 4 h. The value on the *Y*-axis is expressed as log_10_ CFU/mL. The assays were repeated three times with three different biological samples. The error bars represent the means of log_10_ CFU/mL ± SD for each assay. 0.3%, bile salt at 0.3%; 0.02%, bile salt at 0.02%. [MMH594 or mutants/0.02%]-0.03%, *E. faecalis* exposed to 0.02% BS then challenged with 0.03% BS. [MMH594 or mutants/0.02%]-antibiotics, *E. faecalis* exposed to 0.02% BS and then challenged with levofloxacin, vancomycin, or linezolid.

Since BS induced the expression of *ef2593* and *ef0420*, we investigated how *E. faecalis,* if initially exposed to sub-lethal BS concentrations, might adapt to a higher BS level in the human intestine. We pre-exposed *E. faecalis* MMH594 (Fig. 5A), mutant MMH594*ef2593::mariner* (Fig. 5B), and mutant Δ*0420* (Fig. 5C) to 0.02% BS for 15 min and then challenged them with 0.3% BS for 4 h. Pre-exposure increased bacterial survival against 0.3% BS similarly for MMH594 (2 log_10_ CFU/mL), MMH594*ef2592::mariner* (2.35 log_10_ CFU/mL), and Δ*0420* (1.7 log_10_ CFU/mL), suggesting that the absence of single pumps was counterbalanced by other pumps or other resistance mechanisms.

Similarly, we investigated how pre-exposed *E. faecalis* MMH594 might survive high concentrations of antibiotics. We pre-exposed MMH594 to 0.02% BS and then challenged the bacteria with 3× MIC of levofloxacin (Fig. 5D), vancomycin (Fig. 5E), and linezolid (Fig. 5F) for 4 h. Pre-exposure to 0.02% BS increased MMH594 survival against 3× MIC levofloxacin, with a 2-log_10_ increase in CFU/mL. No significant change was observed in MMH594 survivors (±0.02% BS) when challenged with linezolid or vancomycin at a 3× MIC concentration (<0.5 log_10_ CFU/mL).

## DISCUSSION

This study focused on the role of efflux transporters of *E. faecalis* in survival in an environment containing bile salts (BS) at concentrations similar to those of the human gastrointestinal tract, as well as the effects of BS on pump expression and antibiotic resistance. There is limited information about the contribution of *E. faecalis* transporters in efflux-mediated resistance despite their various habitats, which include skin, oral cavity, genitourinary tract, gastrointestinal tract, as well as soil and water, where bacteria often utilize active efflux as a means of survival adaptation (36, 37).

We selected five transporters that were upregulated in *E. faecalis* V583 exposed to BS (EF2592, EF2593) (17), to bovine BS alone or together with SDS (EF0420, EF1814) (19), or from a high-complexity mariner transposon insertion library in *E. faecalis* MMH594 (EF0575) (21). Due to an increase in susceptibility to daptomycin and polymyxin B of the mutant MMH594*ef0575::mariner* (21), we included this candidate in our study despite its not being identified as up-regulated in the long-term bile acid exposure study, as were the cases of EF2592, EF2593, EF0420, and EF1814 (17, 19).

While the *mariner* insertion mutants MMH594*ef2592::mariner* and MMH594*ef2593::mariner* showed the same MICs as those of the wild-type MMH594, *E. faecalis* MMH594 overexpressing EF2592 and EF2593 from a low-copy number plasmid (pAT28) showed a reproducible twofold increase in the MICs of daptomycin and vancomycin. *E. faecalis* transporters cloned into pAT28 yielded a three- to fourfold increase in the corresponding transcript levels. Daptomycin targets the bacterial membrane and has been widely used in treating staphylococcal and enterococcal infections. Our results suggest that EF2592 and EF2593 might provide partial protection of the enterococcal cell membrane from daptomycin action.

Although overexpression of EF2592-EF2593 and EF1814 resulted in modest increases in vancomycin MIC, we do not infer that vancomycin is directly exported by these transporters. Instead, these changes are more likely due to indirect alterations in membrane or cell wall physiology associated with transporter overexpression and bile salt adaptation. Since bile salts perturb the bacterial envelope, activation of membrane-associated transport systems may promote envelope remodeling or stress responses that secondarily influence susceptibility to glycopeptide antibiotics.

Notably, while bile salts induced expression of several transporter genes, the level of induction from the chromosomal loci was apparently insufficient to produce a detectable change in antibiotic MIC in wild-type *E. faecalis*. In contrast, plasmid-mediated overexpression likely exceeded this threshold, revealing the contribution of these transporters to low-level multidrug resistance.

Genetic data of *ef2592* and *ef2593* mutants support the hypothesis that the observed phenotypes require both transporter subunits and are consistent with EF2592-EF2593 functioning as a heterodimeric ABC transporter. Because overexpression of one subunit failed to restore the phenotype in the absence of its chromosomal partner, the observed effects are unlikely to result from independent activity of individual half-transporters. Furthermore, the minimal cross-induction observed between *ef2592* and *ef2593* suggests that the phenotype is not due to transcriptional activation of the partner gene but instead is consistent with increased pairing of the overexpressed subunit with its endogenous counterpart. Future studies will focus on biochemical characterization of EF2592-EF2593 heterodimer assembly, stoichiometry, and transport activity.

The protein EF0575 (239 amino acids) was predicted to be the ATPase component of a two-component ABC transporter and shared conserved residues with EfrC, EfrD, EF2592, and EF2593. More interestingly, a disruption by a *mariner* transposon insertion in the putative ABC transporter EF0575 led to a 15-fold increase in *E. faecalis* susceptibility to linezolid and a 66-fold increase in susceptibility to daptomycin, which were reversed after complementation of the mutant with an intact gene *ef0575* cloned on a plasmid. Furthermore, EF0575 showed homology to the quinupristin-dalfopristin resistance ABC transporter LsaA (EF2720) of *E. faecalis* that is absent in *E. faecium* (10). EF0575, LsaA, and its plasmid-encoded homolog OptrA were involved in *E. faecalis* susceptibility to linezolid (10, 38). Notably, the Lsa protein of *E. faecalis* causes resistance to antibiotics (lincosamides and streptogramin A) through an ABC transporter homolog. Lsa releases antibiotics into the extracellular space, where they are inactivated by either enzymes or the host immune system (10).

Together, these findings support a broader role for ABC transporter systems in intrinsic and adaptive antibiotic tolerance in *E. faecalis*. Although overexpression of EF2592 and EF2593 produced only modest increases in daptomycin and vancomycin MICs, the significant hypersusceptibility of the EF0575 mutant to daptomycin and linezolid demonstrates that membrane-associated ABC transporters can have major protective effects against clinically important antibiotics. The shared homology between EF0575, EF2592, and EF2593, and known resistance determinants, such as LsaA, further suggests that these transporters may function through related mechanisms involving membrane protection and/or active antibiotic efflux. Collectively, these observations highlight ABC transporters as interconnected components of enterococcal stress adaptation and multidrug resistance, particularly against membrane-targeting and protein synthesis–inhibiting antibiotics.

Consistent with this model, ABC transporters EF2592 and EF2593 mediated reproducible two- to fourfold increases in the MICs of five antibiotics from multiple classes, while the MFS efflux pumps, particularly EF1814, produced even stronger effects, with four- to 16-fold increases in resistance to four antibiotics when overexpressed in *L. lactis* NZ9000, underscoring their important contributions to multidrug resistance. Importantly, overexpression of EF0420 and EF1814 in *E. faecalis* OG1RF further increased daptomycin and levofloxacin MICs by four- to eightfold, highlighting the substantial role of these efflux systems in clinically relevant antibiotic resistance phenotypes.

Studies of resistance in enterococci have focused on antimicrobial target alterations for vancomycin and β-lactams. In the case of enterococcal resistance to vancomycin, linezolid and daptomycin are common treatment options (39, 40). The mechanisms of resistance to antibiotics of *E. faecalis* include decreased cell permeability (aminoglycoside), target gene mutations (β-lactams, glycopeptides), ABC efflux pumps (lincosamides), rRNA point mutations (linezolid), or altered membrane-bound protein (daptomycin) (41). In this study, we demonstrated that four additional transporters (two ABC transporters, EF2592 and EF2593, and two MFS transporters, EF0420 and EF1814) individually caused low-level reductions in *E. faecalis* susceptibility to at least one of the four antibiotics (daptomycin, vancomycin, levofloxacin, or oritavancin).

The ATP-binding protein EF0575 did not alter the MIC of *E. faecalis* when overexpressed, and overexpression of both EF0575 and its presumed permease EF0576 produced only a twofold increase in resistance to oritavancin, in contrast to the substantial increases in susceptibility to daptomycin and linezolid found in the *mariner* insertion mutant of EF0575. Taken together, these results suggested that EF0575 together with EF0576 may enable low-level oritavancin resistance, but whether the ATPase of EF0575 functions together with another protein(s) to contribute to daptomycin and linezolid resistance by efflux or another mechanism remains to be determined.

All mutants of the five selected transporters were more affected by BS and DCA than the wild type, which supports the physiologic advantage of target transporters in *E. faecalis* grown in BS-supplemented media. A pre-exposure of the wild type to a sub-lethal concentration of BS (0.02%) before exposure to 0.3% BS allowed the bacteria to increase survival. These data agreed with an increase of at least fourfold in the transcript levels of EF2593 and EF0420. EF2592 and EF2593 might have similar interactions, as were described for EfrCD, and might have a similar function (25).

Thus, ABC transporters EF2592 and EF2593 were more effective in protecting *E. faecalis* against BS, but MFS transporters such as EF0420 and EF1814 were more effective in reducing *E. faecalis* susceptibility to multiple antibiotics. Further investigations are underway to determine the cause of the antibiotic hyper-susceptibility of the mutant of EF0575.

As an example of other microbes that inhabit the gastrointestinal tract and use efflux pumps to survive in the presence of bile salts, *E. coli* uses the AcrAB-TolC efflux pump to survive in the human intestine (42–44). Notably, AcrAB-TolC overexpression also confers resistance to multiple antibiotics (45, 46). The *E. faecalis* manganese exporter MntE also serves as another example of an efflux pump involved in bacterial survival in the human intestine. MntE is a highly conserved transporter that plays a role in manganese homeostasis and virulence in *E. faecalis* and protects the enterococci from excess Mn *in vitro* (47). It is noteworthy that EF0575 was identified as part of an operon regulated by the metalloregulator EfaR that controls the expression of its proteins in a manganese-dependent manner (22). Further investigations are underway to decipher the role of EfaR on the expression of *ef0575*.

In conclusion, we demonstrated that the five candidate transporters, EF2592, EF2593, EF0575, EF0420, and EF1814, identified through BS exposure and *mariner* insertion analyses, contribute to *E. faecalis* survival in the presence of BS while also mediating multidrug resistance phenotypes. These findings raise the possibility that at least part of the observed antibiotic resistance phenotype may represent a physiological consequence of adaptation to the intestinal environment, where exposure to toxic BS selects for transport systems capable of detoxification and membrane protection. Alternatively, these transporters may have evolved broader and more specialized functions that independently support both BS tolerance and antimicrobial resistance. In either case, the overlap between BS adaptation and antibiotic resistance highlights the close relationship between environmental stress responses and clinically relevant resistance mechanisms in *E. faecalis*.

These functions are likely important for *E. faecalis* virulence, gastrointestinal colonization, and persistence across diverse host-associated environments. Although only human (or mammalian)-derived bile acids were tested in this study, enterococci occur in a wide range of hosts with varying homologs of these molecules; thus, some, if not all, of the efflux systems identified in this study likely play similar roles in vertebrate and invertebrate hosts. Given the continued problem of nosocomial transmission of multidrug-resistant enterococci, particularly vancomycin-resistant enterococci (VRE) (4, 48), there is particular interest in developing antimicrobial strategies aimed at intestinal decolonization to reduce both patient reservoirs and transmission. However, our findings suggest that such approaches may face an additional challenge arising from the intestinal milieu itself, where specific BS can induce multiple broad-spectrum efflux systems. This BS-dependent induction may transiently enhance intrinsic tolerance to structurally unrelated antibiotics, potentially reducing antimicrobial efficacy during intestinal colonization and complicating efforts to eradicate VRE from the gastrointestinal tract.

## MATERIALS AND METHODS

### Bacterial strains, media, and growth conditions

Bacterial strains, plasmids, and primers used in this study are listed in Table 5. Daptomycin, levofloxacin, tetracycline, linezolid, oritavancin, vancomycin, bile salt (BS; composition of BS = 50% CA/50% DCA), cholate (CA), deoxycholate (DCA), and spectinomycin were purchased from Sigma-Aldrich (St. Louis, MO). *E. faecalis* and *L. lactis* NZ9000 containing plasmid pAT28 (49), pMSP3535 (35), and various derived constructs were grown at 37°C in brain heart infusion (BHI) supplemented with spectinomycin at 750 μg/mL (pAT28) or erythromycin at 10 μg/mL (pMSP3535) (50). All other *E. faecalis*, wild type and mutants, were grown in BHI medium unless otherwise stated. Bacteria were cultured under conditions as previously reported by Shepard and Gilmore (51). In brief, fresh culture was initiated from an overnight culture (1:100 dilution) in pre-warmed BHI medium and cultured at 37°C without shaking. Bacterial growth was monitored using a spectrophotometer at an optical density (OD) of 600 nm, and CFU/mL was determined by plate counting (at OD_600_ = 0.3; CFU/mL = 2.2 10^8^) (50, 52). The assays were repeated at least three times to verify the high viable count obtained under nutrient-rich growth conditions. All *E. faecalis* strains were cultured and quantified using identical conditions.

**TABLE 5:** Bacterial strains, plasmids, and primers used in this study

Strain, plasmid, or primer	Genotype or relevant characteristic(s)^*a*^	Reference or source
*E. faecalis*		
MMH594	*Enterococcus faecalis* (strain EnGen0310)	(53, 54)
MMH594*ef2592::mariner*	*Mariner* insertion mutant	(21)
MMH594*ef2593::mariner*	*Mariner* insertion mutant	(21)
MMH594*ef0575::mariner*	*Mariner* insertion mutant	(21)
Δ*0420*	In-frame deletion mutant	This study
MMH594(pAT28)	Empty plasmid, spectinomycin^R^	This study
MMH594(pAT28::*ef2592*)	EF2592-overexpressor, (spectinomycin^R^)	This study
MMH594(pAT28::*ef2593*)	EF2593-overexpressor, (spectinomycin^R^)	This study
MMH594(pAT28::*ef0575*)	EF0575-overexpressor, (spectinomycin^R^)	This study
MMH594(pAT28::*ef0420*)	EF0420-overexpressor, (spectinomycin^R^)	This study
MMH594(pAT28::*ef1814*)	EF1814-overexpressor, (spectinomycin^R^)	This study
MMH594(pAT28::*ef2592-ef2593*)	EF2592-EF2593-double overexpressor, (spectinomycin^R^)	This study
MMH594(pAT28::*ef0575-ef0576*)	EF0575-EF0576-double overexpressor, (pAT28, spectinomycin)^R^	This study
MMH594*ef2592::mariner*(pAT28::*ef2592*)	EF2592-overexpressor in mutant, (spectinomycin^R^)	This study
MMH594*ef2592::mariner*(pAT28::*ef2593*)	EF2593-overexpressor in mutant, (spectinomycin^R^)	This study
MMH594*ef2593::mariner*(pAT28::*ef2592*)	EF2592-overexpressor in mutant, (spectinomycin^R^)	This study
MMH594*ef2593::mariner*(pAT28::*ef2593*)	EF2593-overexpressor in mutant, (spectinomycin^R^)	This study
MMH594*ef0575::mariner*(pAT28::*ef0575*)	EF0575-overexpressor in mutant, (spectinomycin^R^)	This study
OG1RF	*Enterococcus faecalis* (virulent, plasmid-free strain)	(34)
OG1RF(pMSP3535)	Empty plasmid, erythromycin^R^	This study
OG1RF(pMSP3535::*ef0420*)	EF0420-overexpressor, (erythromycin^R^)	This study
OG1RF(pMSP3535::*ef1814*)	EF1814-overexpressor, (erythromycin^R^)	This study
*L. lactis*		
NZ9000	*Lactococcus lactis* Expression Systems	Boca Scientific
NZ9000(pMSP3535)	Empty plasmid, erythromycin^R^	This study
NZ9000(pMSP3535::*ef2592*)	EF2592-overexpressor, (erythromycin^R^)	This study
NZ9000(pMSP3535::*ef2593*)	EF2593-overexpressor, (erythromycin^R^)	This study
NZ9000(pMSP3535::*ef0576*)	EF0576-overexpressor, (erythromycin^R^)	This study
NZ9000(pMSP3535::*ef0575-ef0576*)	EF0575-0576-double overexpressor, (erythromycin^R^)	This study
NZ9000(pMSP3535::*ef0420*)	EF0420-overexpressor, (erythromycin^R^)	This study
NZ9000(pMSP3535::*ef1814*)	EF1814-overexpressor, (erythromycin^R^)	This study
*E. coli*		
DH5α	F^−^ Φ80*lac*ZΔM15 Δ(*lac*ZYA-*arg*F) U169 *rec*A1 *end*A1	Life Technology
Plasmids		
pLT06	Engineering in-frame deletion in *E. faecalis* mutant	
pAT28	Broad-range shuttle vector, spectinomycin^R^	(49)
pMSP3535	Gram + shuttle vector, nisin-inducible expression, erythromycin^R^	Addgene
Primers for real-time RT-PCR assays		
*recA* (internal control)	Forward 5′ GCAACGAAATGGTGGAACAG 3′	(55)
	Reverse 5′ AAGGCATCGGCAATCTCTAAG 3′	
EF2592	Forward 5′ ACAGGTGCTGGGAAAACAAC3′	This study
	Reverse 5′ ACTTCTTCTTCGCTCGCTTG 3′	
EF2593	Forward 5′ GGACTGGTTCTGGGAAAACA 3′	This study
	Reverse 5′ AGGCTTGCCAAATTTCTTCA 3′	
EF0575	Forward 5' AGGCTTTGGAAAAGGTCGAT 3′	This study
	Reverse 5' AAATCCGCATTTTGAGCAAG 3′	
EF0420	Forward 5′ GATTCCCATCAGTGCTTGGT 3′	This study
	Reverse 5′ GCGGGAAAATTGAAAGCATA 3′	
EF1814	Forward 5′ ATGTGGCGTTACCATCCATT 3′	This study
	Reverse 5′ TAGAACCACCAATCCCTTGC 3′	
Primers for *E. faecalis* efflux pump overexpressors		
Cloned into plasmid pAT28		
EF2592-(EcoRI)-F	Forward 5′ ATATGAATTCATGAGTAGTCGACCAAAA 3′	This study
EF2592-(XbaI)-R	Reverse 5′ ATATTCTAGATTAGGCTACTTCTTCTGAAA 3′	This study
EF2593- (EcoRI)-F	Forward 5′ ATATGAATTCATGTCATTATGGTCGGTCTT 3′	This study
EF2593-(XbaI)-R	Reverse 5′ ATATTCTAGATCATGCAAGGTCCTCC 3′	This study
EF0575- (EcoRI)-F	Forward 5′ ATGCGAATTCATGTTAGAAGTGAAAA 3′	This study
EF0575-(BamHI)-R	Reverse 5′ ATATGGATCCTTATTCCCCCTCTCCA 3′	This study
EF-0576-(XbaI)-R	Reverse 5′ ATATTCTAGATCATAACGATCTCTC 3′	This study
EF0420-(EcoRI)-F	Forward 5′ATATGAATTCATGCAATTTAATTTGAAACTTACA 3′	This study
EF0420-(XbaI)-R	Reverse 5′ ATATTCTAGATTATTTCATTGCACCTACTT 3′	This study
EF1814-(EcoRI)-F	Forward 5′ ATATGAATTCATGATCGATAGAAAAAAAGT 3′	This study
EF1814-(XbaI)-R	Reverse 5′ ATATTCTAGATTAAAAATAAAACCTAACTC 3′	This study
Cloned into plasmid pMSP3535		
EF2592-(SapI)-F	Forward 5′ ATATGCTCTTATGAGTAGTCGACCAAAA 3′	This study
EF2592-(XbaI)-R	Reverse 5′ ATATTCTAGATTAGGCTACTTCTTCTGAAA 3′	This study
EF2593- (SapI)-F	Forward 5′ ATATGCTCTTATGTCATTATGGTCGGTCTT 3′	This study
EF2593-(XbaI)-R	Reverse 5′ ATATTCTAGATCATGCAAGGTCCTCC 3′	This study
EF0575- (SapI)-F	Forward 5′ ATGCGCTCTTATGTTAGAAGTGAAAA 3′	This study
EF-0576-(SapI)-F	Forward 5′ ATGCGCTCTTATGTTAGAAGTGAAAA 3′	This study
EF-0576-(XbaI)-R	Reverse 5′ ATATTCTAGATCATAACGATCTCTC 3′	This study
EF0420-(SapI)-F	Forward 5′ ATATGAATTCATGCAATTTAATTTGAAACTTACA 3′	This study
EF0420-(XbaI)-R	Reverse 5′ ATATTCTAGATTATTTCATTGCACCTACTT 3′	This study
EF1814-(SapI)-F	Forward 5′ ATATGCTCTTATGATCGATAGAAAAAAAGT 3′	This study
EF1814-(XbaI)-R	Reverse 5′ ATATTCTAGATTAAAAATAAAACCTAACTC 3′	This study
Primers for Δ*0420* mutant		
EF0420-F1-BamHI	Forward 5′ GGTGGTGGATCCGATTGCCGTTGATGCCGTC 3′	This study
EF0420-R1-SalI	Reverse 5′ CGGGTCGACCGTACAAAATGTTCCGATTAG 3′	This study
EF0420-F2-XhoI	Forward 5′ CGGCTCGAGTCACGGGACTGCTGTAAAC 3′	This study
EF0420-R2-PstI	Reverse 5′ GTTGTTCTGCAGCTTGGGACATTGATGGATTC 3′	This study
OriF	Forward 5′ CAATAATCGCATCCGATTGCA 3′	(56)
KS05seqR	Reverse 5′ CCTATTATACCATATTTTGGAC 3′	(56)

^*a*^ Restriction sites are underlined.

### Antibiotic susceptibility assay

The MICs of *E. faecalis* were determined by broth microdilution at 37°C for 24 h in Mueller-Hinton broth (MHB) (Thermo Fisher Scientific, Bedford, MA) with an initial inoculum of ~5 × 10^5^ CFU/mL, as previously described and by the CLSI standard (57, 58). *E. faecalis* was diluted 100-fold and inoculated into microtiter plates (Fisher Scientific, Pittsburgh, PA) containing twofold serial dilutions of antibiotics or BS. For co-exposure, 0.02% or 0.1% BS was added to the MHB medium, together with antibiotics, and the MIC was determined as described. MIC was the lowest drug concentration that produced no visible turbidity after incubation at 37°C for 24 h. The MICs of daptomycin were determined using methods described by Chuang et al. (59).

### Growth in the presence of BS or cholates

*E. faecalis* wild type, efflux transporter mutants, and overexpressors were cultured overnight at 37°C in BHI or BHI supplemented with 750 μg/mL of spectinomycin (plasmid pAT28 constructs) (56). The BS exposure assays were carried out as described by Repoila et al. (52). The overnight cultures were diluted 1:500 in pre-warmed BHI medium and grown to an OD_600_  ~ 0.3 (2.2 × 10^8^ CFU/mL) without shaking. Each *E. faecalis* culture was divided equally and treated with either 0.1%–0.3% BS, 0.1%–0.2% DCA, or 0.1%–0.2% CA. Samples were collected at 24 h, and colony counts were performed to determine the CFU/mL for each condition. A subculture of *E. faecalis* was used as a control and was cultured without BS.

### BS adaptation and challenge assays

*E. faecalis* wild type and efflux transporter mutants (OD_600_  ~ 0.3; 2.2 × 10^8^ CFU/mL) were exposed to a sub-lethal concentration of BS (0.02%) for 15 min, as described by Zhang et al. (24). The adapted *E. faecalis* were collected by centrifugation and re-suspended in BHI supplemented with 0.3% BS and regrown for 4 h at 37°C without shaking. In parallel, a culture of *E. faecalis* was grown directly in BHI-0.3% BS and served as a non-adaptation control. CFU/mL of all strains was determined by colony counting on BHI agar plates.

To perform *E. faecalis* adaptation to 0.02% BS and antibiotic challenge assays, we followed the same procedure for adaptation as described above (0.02% BS, 15 minutes exposure). The adapted *E. faecalis* were collected by centrifugation and re-suspended in BHI supplemented with threefold the MICs of levofloxacin, linezolid, or vancomycin, and regrown for 4 h at 37°C without shaking. In parallel, a culture of *E. faecalis* was grown in the presence of the same concentrations of antibiotics and served as unexposed controls.

### Induction assays and quantitative real-time RT-PCR

*E. faecalis* (OD_600_ ~0.4) was cultured in the presence of 0.1% or 0.02% BS for 60 min to induce the expression of *E. faecalis* efflux pumps. RNA extraction and real-time qRT-PCR assays were done as previously described by Ruiz-Cruz et al. (55). Samples were collected, resuspended in buffer L (10 mM Tris-HCl, pH 8.0, 1 mM EDTA, 1 mg/mL lysozyme, 160 units/mL mutanolysin), and then incubated at 37°C for 10 min. Total RNA was extracted using the RNeasy mini-Kit (QIAGEN), and an additional DNase I digestion was performed. RNA concentration was determined using a NanoDrop ND-1000 Spectrophotometer (Thermo Fisher Scientific, Bedford, MA). Primers designed for the RT-PCR assays were synthesized at Eton Bioscience Inc. (Eton Bioscience, Boston, MA) and are listed in Table 5. cDNAs were synthesized using the iScript cDNA synthesis kit (Bio-Rad, Hercules, CA), followed by real-time RT-PCR assays using SYBR Green Supermix (Bio-Rad, Hercules, CA) and the CFX Opus 96 Real-Time PCR system (Bio-Rad, Hercules, CA). The housekeeping gene *recA* was used as an internal control for qRT-PCR normalization, as previously described by Zheng et al. (60).

Since this study examined transcriptional responses to bile salt exposure rather than DNA-damaging conditions, *recA* was selected as the reference gene for normalization. The stability of *recA* expression was experimentally verified under the bile salt exposure conditions used in this study, with relative transcript levels remaining unchanged following exposure to 0.02% or 0.1% bile salts for 1 hour (Table S1).

### Construction of *E. faecalis* overexpressing efflux transporters

We amplified DNA fragments that carried the corresponding transporter genes (*ef2592*, *ef2593*, *ef0575*, *ef0420, ef1814*) using primers listed in Table 5. The primers were designed based on the DNA sequence of the published genome of *E. faecalis* V583 (29). The PCR product and the plasmid pAT28 were digested with appropriate restriction enzymes, as shown in Table 5, and then ligated to generate the plasmid constructs pAT28::*ef2592*, pAT28::*ef2593*, pAT28::*ef0575*, pAT28::*ef0420*, and pAT28::*ef1814*. The plasmid constructs were introduced into *E. coli* DH5α and selected on BHI agar plates supplemented with spectinomycin at 150 µg/mL. Plasmid constructs were re-extracted and then introduced into *E. faecalis* MMH594, and transformants were selected on BHI agar plates supplemented with spectinomycin at 750 µg/mL. DNA sequencing was performed to verify the correct DNA sequences of the *E. faecalis* transporters.

### Construction of *E. faecalis* MMH594 overexpressing efflux transporters (EF2592-EF2593) and (EF0575-EF0576)

We amplified a DNA fragment that carried the corresponding transporter genes *ef2592-ef2593* using primers EF-2592-SapI and EF-2593-XbaI (Table 5), then cloned the PCR product into plasmid pAT28, followed by transformation into *E. coli* DH5α, and selected the correct transformants on BHI plus spectinomycin at 150 µg/mL. Plasmid pAT28 carries a *lac* promoter that drives the expression of the cloned structural genes, and pAT28 uses the replicon of plasmid pAMβ1, which has a copy number of 5–10 (49). Plasmid construct pAT28::*ef2592-ef2593* was re-extracted from *E. coli* DH5α and transformed into *E. faecalis* MMH594, and the transformant was selected on BHI agar plates supplemented with spectinomycin at 750 µg/mL. The *ef2592-ef2593* sequence was verified by DNA sequencing and compared with the published genome of *E. faecalis* V583 and MMH594 (29).

We followed the same protocol and amplified a DNA fragment that carried the two adjacent genes *ef0575* and *ef0576* using primers EF-0575-EcoRI and EF-0576-XbaI (Table 5) and then cloned the PCR product into plasmid pAT28. The plasmid construct pAT28::*ef0575-ef0576* was introduced into *E. coli* DH5α and selected on BHI agar plates supplemented with spectinomycin at 150 µg/mL. The plasmid construct was re-extracted and then introduced into *E. faecalis* MMH594, and the transformant was selected on BHI agar plates supplemented with spectinomycin at 750 µg/mL. DNA sequencing was performed to verify the correct DNA sequences of the *E. faecalis ef0575-ef0576*.

### Construction of *E. faecalis* OG1RF overexpressing efflux transporters EF0420 and EF1814

We followed the same protocol and amplified a DNA fragment that carried the genes *ef0420* and *ef1814* using primers listed in Table 5 and then cloned the PCR products into the nisin-inducible plasmid pMSP3535 (35). The plasmid constructs pMSP3535::*ef0420* and pMSP3535::*ef1814* were introduced into *E. coli* DH5α and selected on BHI agar plates supplemented with erythromycin at 300 µg/mL. The plasmid construct was re-extracted and then introduced into *E. faecalis* OG1RF, and the transformant was selected on BHI agar plates supplemented with erythromycin at 10 µg/mL. DNA sequencing was performed to verify the correct DNA sequences of the *E. faecalis ef0420* and *ef1814*. To induce the overexpression of *ef0420* and *ef1814*, we added 25 ng/mL of nisin to the bacterial cultures as recommended by the manufacturer (Addgene) (35). The MICs were performed as described above in the presence or absence of nisin.

### Construction of *E. faecalis* deletion mutant Δ*0420*

A deletion mutant of *E. faecalis* MMH594 lacking the *ef0420* gene was generated by allelic replacement, as previously described by Thurlow et al. (56). DNA fragments of 962 bp upstream and 907 bp downstream of the *ef0420* locus were amplified by PCR using primer pairs F1 up-forward/R1 up-reverse and F2 down-forward/R2 down-reverse (Table 5). These fragments were fused by overlap extension PCR to produce a 1,869-bp product. The final PCR product was digested with *Bam*HI and *Pst*I and then ligated into the pLT06 plasmid pre-digested with the same enzymes using T4 DNA ligase (NEB). The recombinant plasmid was transformed into *E. coli* EC1000 for propagation and sequence verification. After confirmation by PCR using OriF and KS05seqR primers, the plasmid construct was introduced into *E. faecalis* MMH594 via electroporation. Allelic exchange was carried out using temperature shifts and p-chlorophenylalanine counterselection, as previously described (56). Candidate colonies were screened by PCR using EF0420-F1 and EF0420-R2 primers (Table 5), and the deletion was confirmed by Sanger sequencing.

### Construction of *L. lactis* NZ9000 overexpressing efflux transporters

*L. lactis* NZ9000 was purchased from Boca Scientific (Dedham, MA), and competent cells were prepared following the specific protocol for *L. lactis* NICE expression system (MoBiTec GmbH, Biozol, Germany). In brief, NZ9000 was cultured overnight at 30°C in M17 broth (Fisher Scientific, Bedford, MA) supplemented with 0.5 M sucrose, 2.5% glycine, and 0.5% lactose without shaking. Plasmid pMSP3535 constructs carrying *E. faecalis* genes *ef2592*, *ef2593, ef0576*, *ef0575-0576*, *ef0420*, and *ef1814* were generated using the same protocol and primers listed in Table 5. Bacteria were transformed by electroporation with plasmid pMSP3535 or pump constructs, and transformants were cultivated in M17-glucose medium supplemented with MgCl2 (20 mM) and CaCl2 (2 mM). NZ9000 transformants were selected on M17-glucose plates supplemented with erythromycin at 10 µg/mL. To perform MIC assays, fresh cultures were initiated from overnight cultures and grew without shaking at 30°C until OD_600_ reached 0.5. MICs of *L. lactis* were carried out by broth microdilution at 30°C for 16–24 h in M17-glucose (Fisher Scientific, Bedford, MA) ±25 ng/mL of nisin. The MIC is determined as the lowest antibiotic concentration that prevents bacterial growth (13).

### Statistical analysis

The experiments were performed in triplicate with three independent biological samples. The data were expressed as a mean ± SD. The data were analyzed using one-way analysis of variance (ANOVA). The pairwise comparison was performed using a *t*-test to compare sample groups and combined with a Bonferroni adjustment. The threshold for significance was set at *P*-value < 0.05.

## SUPPLEMENTAL MATERIAL

Table S1 msphere.00489-26-s0001.docx Stability of *recA* transcript levels in *E. faecalis* following exposure to bile salts.

ASM does not own the copyrights to Supplemental Material that may be linked to, or accessed through, an article. The authors have granted ASM a non-exclusive, world-wide license to publish the Supplemental Material files. Please contact the corresponding author directly for reuse.
