# Supplementary material for: ABC and MFS efflux transporters mediate antibiotic resistance and bile salt tolerance in Enterococcus faecalis
Source: mSphere. 2026 Sep 15;11(10):e00489-26. doi: 10.1128/msphere.00489-26 (PMC13711829; doi:10.1128/msphere.00489-26)
Supplement: Supplementary file 1 [file msphere.00489-26-s0001.docx]

**ABC and MFS Efflux Transporters Mediate Antibiotic Resistance and Bile Salt Tolerance in *Enterococcus faecalis***

Truong-Bolduc Q.C., Wang Y., Mello S.S., Phillips M.C., Gilmore M.S., and Hooper D.C.

**Supplementary data**

**Supplementary Data**

**Table S1.** Stability of *recA* transcript levels in *E. faecalis* following exposure to bile salts.

|  | **Relative *recA* transcript level (FC)*** | | |
| --- | --- | --- | --- |
| ***E. faecalis*** | BS (0%)** | BS (0.02%)** | BS (0.1%)** |
| MMH594 | 1±0.01 | 1.01±0.01 | 0.99±0.01 |
| MMH594(pAT28) | 1±0.01 | 1±0.01 | 1.01±0.01 |
| MMH594(pAT28::*ef2592*) | 1±0.01 | 1±0.01 | 1.02±0.02 |
| MMH594(pAT28::*ef2593*) | 1±0.01 | 0.99±0.01 | 1±0.01 |
| MMH594(pAT28::*ef0575*) | 1±0.01 | 1±0.01 | 1±0.02 |
| MMH594(pAT28::*ef0420*) | 1±0.01 | 1±0.01 | 1.01±0.01 |
| MMH594(pAT28::*ef1814*) | 1±0.01 | 1.02±0.01 | 1.03±0.02 |
| MMH594(pAT28::*ef2592-ef2593*) | 1±0.01 | 1.03±0.02 | 1.01±0.01 |
| MMH594(pAT28::*ef0575-ef0576*) | 1±0.01 | 0.98±0.02 | 1±0.01 |
| * *E. faecalis* strains were exposed to BS for 1 hour | | | |
| **BHI medium with or without bile salt (BS) | | | |
| *E. faecalis* MMH594 grown in BHI without bile salts (0%) was used as the reference condition. | | | |
